# Creating Live Interactions to Mitigate Barriers (CLIMB): A Mobile Intervention to Improve Social Functioning in People With Chronic Psychotic Disorders

**DOI:** 10.2196/mental.6671

**Published:** 2016-12-13

**Authors:** Bruno Biagianti, Danielle Schlosser, Mor Nahum, Josh Woolley, Sophia Vinogradov

**Affiliations:** ^1^ Department of Psychiatry University of California, San Francisco San Francisco, CA United States; ^2^ Department of Psychiatry University of Milan Milano Italy; ^3^ Posit Science, Inc. San Francisco, CA United States; ^4^ Hebrew University Jerusalem Israel; ^5^ Department of Psychiatry University Of Minnesota Minneapolis, MN United States

**Keywords:** psychosis, social cognition, digital health, mobile health

## Abstract

**Background:**

Numerous psychosocial interventions for individuals with chronic psychotic disorders (CPD) have shown positive effects on social cognitive and functional outcome measures. However, access to and engagement with these interventions remains limited. This is partly because these interventions require specially trained therapists, are not available in all clinical settings, and have a high scheduling burden for participants, usually requiring a commitment of several weeks. Delivering interventions remotely via mobile devices may facilitate access, improve scheduling flexibility, and decrease participant burden, thus improving adherence to intervention requirements. To address these needs, we designed the Creating Live Interactions to Mitigate Barriers (CLIMB) digital intervention, which aims to enhance social functioning in people with CPD. CLIMB consists of two treatment components: a computerized social cognition training (SCT) program and optimized remote group therapy (ORGT). ORGT is an innovative treatment that combines remote group therapy with group texting (short message service, SMS).

**Objectives:**

The objectives of this single-arm study were to investigate the feasibility of delivering 6 weeks of CLIMB to people with CPD and explore the initial effects on outcomes.

**Methods:**

Participants were recruited, screened and enrolled via the Internet, and delivered assessments and interventions remotely using provided tablets (iPads). Participants were asked to complete 18 hours of SCT and to attend 6 remote group therapy sessions. To assess feasibility, adherence to study procedures, attrition rates, engagement metrics, and acceptability of the intervention were evaluated. Changes on measures of social cognition, quality of life, and symptoms were also explored.

**Results:**

In total, 27 participants were enrolled over 12 months. Remote assessments were completed successfully on 96% (26/27) of the enrolled participants. Retention in the 6-week trial was 78% (21/27). Of all the iPads used, 95% (22/23) were returned undamaged at the end of the intervention. Participants on average attended 84% of the group therapy sessions, completed a median of 9.5 hours of SCT, and posted a median of 5.2 messages per week on the group text chat. Participants rated CLIMB in the medium range in usability, acceptability, enjoyment, and perceived benefit. Participants showed significant improvements in emotion identification abilities for prosodic happiness (*P*=.001), prosodic happiness intensity (*P*=.04), and facial anger (*P*=.04), with large within-group effect sizes (*d*=.60 to *d*=.86). Trend-level improvements were observed on aspects of quality of life (*P* values less than .09). No improvements were observed for symptoms.

**Conclusions:**

It is feasible and acceptable to remotely deliver an intervention aimed at enhancing social functioning in people with CPD using mobile devices. This approach may represent a scalable method to increase treatment access and adherence. Our pilot data also demonstrate within-group gains in some aspects of social cognition after 6 weeks of CLIMB. Future randomized controlled studies in larger samples should evaluate the extent to which CLIMB significantly improves social cognition, symptoms, and quality of life in CPD.

## Introduction

Individuals with chronic psychotic disorders (CPD) struggle with poor social functioning, namely in their ability to engage in social interactions and to create meaningful relationships [1]. Contributors to poor social functioning in CPD include pervasive impairments in social cognition [2], including the perception, interpretation and processing of socially-relevant information [3]. For example, individuals with CPD show impairments in gaze perception [4], emotion perception [5,6], social cue perception [7], theory of mind [8], attribution style [9] and empathy [10]. Impairments across all of these core domains of social cognition persist throughout the course of the illness and have been linked to low occupational status, impaired community functioning, reduced capabilities for independent living, high relapse rates and reduced quality of life [1,11-14]. As a result, integrating treatments for social cognition into psychosocial interventions may be critical to improving social functioning for individuals with CPD [15].

Among the various interventions developed to improve social cognition over the past two decades (for a review, see [16]), integrated psychological therapy (IPT) [17] and cognitive enhancement therapy (CET) [18] are two models that emphasize that treatments for social cognition should take place within a meaningful social context so that patients can practice the trained social cognitive abilities in supervised real-world social situations [19]. The goal of this approach is to increase the likelihood of skill transfer to everyday life settings and to promote successful participation in real-world social situations. For example, in CET, computer-based training of social cognition is integrated with group work on social skills development. In these weekly groups, patients with CPD practice structured social interactions, solve real-life social dilemmas, do appraisal of affect and social contexts, initiate and maintain conversations and receive feedback from other patients and coaches. Integrating the group sessions has been shown to be critically important for CET to generalize to real-life accomplishments in social and occupational roles [18], although a recent study suggests that combining elements of computer-based cognitive training and social skills groups did not induce greater benefits than cognitive training alone [20]. Finally, group-based interventions are known to sustain engagement and to reduce stigma and isolation [21-23].

Research overall demonstrates positive effects of these group-based integrated interventions on social cognitive and functional outcome measures [17,18]. Unfortunately, several factors limit access to and engagement with these treatments. First, many of these interventions are currently offered in only a few specialized programs, and may not be accessible to people who live in rural or under-resourced areas [24]. Second, these programs require specially trained therapists, who may not be available in all clinical settings [25]. Third, these interventions have a high scheduling burden, usually requiring a commitment of several months (and up to two years), in-person weekly visits to clinics and the organization of patient groups for program delivery. This scheduling burden can become untenable for people who are employed, have caregiver demands, have other responsibilities to manage or are without transportation. Lastly, some individuals with CPD hesitate to approach traditional mental health treatment settings because of stigma, which interferes with help-seeking behaviors [26].

Recent advances in digital technology and mobile platforms can help overcome these limitations by supporting the delivery of interventions remotely to individuals with CPD who are unable or unwilling to come in to the clinic, and do not own or have easy access to Internet-connected computers. Mobile interventions offer several benefits compared to in-person approaches. First, they enable scheduling flexibility and decrease scheduling burdens, thus facilitating accessibility and compliance with intervention requirements and ultimately increasing cost-effectiveness [27]. Second, digital technology can enrich the quality of treatment by incorporating innovative methods of communication, and by making treatment adaptive and responsive to dynamic, ecologically valid, real-time data [28]. Third, mobile interventions delivered in real-time may be accessed with greater frequency than in-person treatment approaches for brief therapeutic interactions that help consolidate support and maintain inter-session continuity [29]. Fourth, delivering the intervention in real-world settings may support the retention, reinforcement and successful generalization of trained skills [30]. Finally, mobile interventions can include opportunities for remote social engagement, like text-based motivation coaching from trained therapists or social networking via direct peer-to-peer messaging [30].

Guided by these principles, we designed Creating Live Interactions to Mitigate Barriers (CLIMB), a mobile psychosocial intervention that aims to enhance social functioning in people with CPD. CLIMB consists of the following two treatment components: (1) computerized social cognition training (SCT) exercises, and (2) optimized remote group therapy (ORGT). In line with the principles of IPT and CET, we opted for a hybrid approach, blending a structured training of social cognitive abilities (SCT) with an intervention that combines weekly group teletherapy with group texting (short message service, SMS) (ORGT). The principal goal of CLIMB is to enhance social functioning by driving improvements in social cognition, quality of life and clinical symptoms. However, in this open-label pilot study, our main objective is to validate the feasibility of the mobile intervention approach in people with CPD. Over the course of 12 months, we delivered CLIMB for 6 weeks via loaned tablets (iPads) to 27 participants recruited remotely from various locations in the United States and Canada. We evaluated adherence with study procedures, attrition rates, engagement metrics and acceptability of the intervention. In addition, we explored the effects of 6 weeks of CLIMB on measures of social cognition, quality of life and clinical symptoms. Finally, we explored possible predictors of engagement with treatment components, and examined whether engagement influenced changes in outcome measures.

## Methods

### Intervention Design

CLIMB consists of novel treatment components consisting of SCT exercises and ORGT.

#### Social Cognitive Training Exercises

The SCT computerized exercises used in the study are a subset of the social cognitive training suite called SocialVille, developed by Nahum et al, which aims to treat social cognition deficits targeting the impaired brain systems underlying social cognition [31]. The rationale for the training exercises has been reported elsewhere [31]. Briefly, the exercises harness the principles of brain plasticity, employing speeded, accurate and increasingly more challenging discriminations of socially-relevant information (eg, eye gazes, emotional faces, prosody, social situations). Participants progress through each exercise in a defined order of difficulty, generally moving from more simple levels (eg, easy to discriminate stimulus types, less response options) to more complex levels (eg, greater rule complexity, greater similarity between stimuli, etc). A single-arm open-label feasibility study of SocialVille delivered remotely to a small sample of young adults with psychosis found high adherence with the training requirements, and significant improvements on untrained measures of social cognition, social functioning, motivation and reward sensitivity [31].

The SCT exercises chosen in CLIMB target gaze perception, visual emotion perception, prosody, theory of mind, affective memory and attribution bias, as these core domains of social cognition are pervasively impaired in CPD and underlie most critical factors of real-world functioning including decreased quality of life and poorer community and occupational functioning [1,11-14]. A full description of the exercises is provided in Multimedia Appendix 1. The exercises are personalized, in that (1) the level of training difficulty and progression for each exercise is individually adaptive to ensure that each user is appropriately challenged; (2) although the total number of levels to be completed for each exercise is fixed and equal for all participants, they can choose any 4 of the 7 exercises to complete on every session; (3) participants can dynamically set the desired number of sessions to complete every week and monitor their progress in real-time; and (4) summary screens including game metrics (points) and exercise metrics (progress) are shown to the participant at the end of each level.

#### Optimized Remote Group Therapy

ORGT is an innovative integrated treatment approach that uses mobile technology to implement weekly group teletherapy sessions with group texting. In line with current recommendations for group therapy in CPD, individuals are grouped into cohorts of 3 to 6, within a similar age range (within 5 years) [32,33]. A master’s level clinician leads the group teletherapy sessions and a moderator assists participants during ORGT by facilitating and organizing the sessions and by leading the group text chat.

##### Group Teletherapy Sessions

Participants attend weekly, 60-minute group teletherapy sessions. Sessions are based on recovery-model principles [34] and on the Raise Early Treatment Program Manual [35]. Prior to beginning the first group therapy session, two online surveys that assess current social difficulties and preference for topics to be discussed during the group teletherapy sessions are administered. The clinician evaluates data from these surveys to familiarize herself with the patient-centered goals. In the first session, after introductions, the clinician teaches Specific, Meaningful, Agreed Upon, Realistic, Timely (SMART) goals [36]. For every session, participants set a SMART goal appropriate to their level of recovery. The following sessions start with an initial check-in (approximately 10 minutes) where participants report on the SMART goal that they attempted during the week. This is followed by psycho-education and a discussion of shared experiences (approximately 15 minutes). Here, the clinician lets participants pick a topic. The topics covered include how to make friends, how to improve social skills, how to improve motivation, how to identify a relapse and how to succeed in a job or at school. Participants discuss the topic while the clinician moderates the conversation and invites participation by all members of the group. The clinician also contributes by sharing information, such as feedback on the importance of reciprocity in social relationships or ways to motivate yourself with rewards. The next segment of the session is dedicated to learning or practicing a skill (approximately 15 minutes). The skills covered include a variety of mindfulness techniques, social skills training and relapse prevention planning. Finally, participants set a personalized SMART goal for the upcoming session (approximately 10 minutes).

##### Group Text Chat

The group text chat is used between video calls to optimize group teletherapy by maintaining inter-treatment session continuity with participants, by helping engage them in study procedures and by offering more opportunities for social engagement and peer support. The clinician and the moderator use the group text chat to (1) supplement group teletherapy sessions by sending links and articles about information and topics discussed during the video calls; (2) notify the group of study updates; (3) remind the group of scheduled sessions and training requirements; and (4) message participants for remote technical support and solution-focused problem-solving. The moderator encourages participants to use the group text chat to share personal artistic projects with the group through links, videos, pictures, drawings, poems and quotes. The clinician and moderator promote a non-stigmatizing approach to psychotic experiences.

### Feasibility Trial

#### Recruitment and Enrollment

For the pilot feasibility trial, study participants were recruited online: information about the study aims and procedures was posted on Craigslist, Reddit, the National Alliance on Mental Illness newsletter and our study website. Interested individuals contacted research staff via phone call, text or email. During the initial phone screening, study personnel verified that potential participants met the following inclusion criteria: (1) prior clinical diagnosis of schizophrenia, schizoaffective disorder or bipolar disorder with psychotic features, (2) age 18 to 65 years, (3) no neurological disorder or history of traumatic brain injury, (4) no substance dependence or serious substance use in the past 6 months, (5) current treatment with a mental health care provider, (6) no hospitalization in the last 3 months and no changes in psychiatric medications for at least 1 month, (7) visual, auditory and motor capacity to use an iPad, and (8) access to an Internet-connected device with a camera/webcam and an active email account. This last criterion was required in order to sign the consent form digitally and to undergo the eligibility diagnostic interview before iPad shipment.

After the initial phone screening, study personnel provided informed consent documents to participants remotely using Qualtrics (Provo, Utah, USA). Following the initial screening and consent, participants underwent a diagnostic evaluation using the Structured Clinical Interview for the Diagnostic and Statistical Manual of Mental Disorders-IV Text Revision (DSM-IV-TR) (SCID) [37], which was administered remotely by graduate-level psychology students, using the Health Insurance Portability and Accountability Act (HIPAA) compliant video-calling software Vsee (Vsee Lab, LLC, Sunnyvale, California, USA). Participants who met DSM-IV-TR criteria for schizophrenia, schizoaffective disorder or bipolar disorder (psychotic disorders) with psychotic features were enrolled in the study.

#### Study Procedures

**Figure 1:**
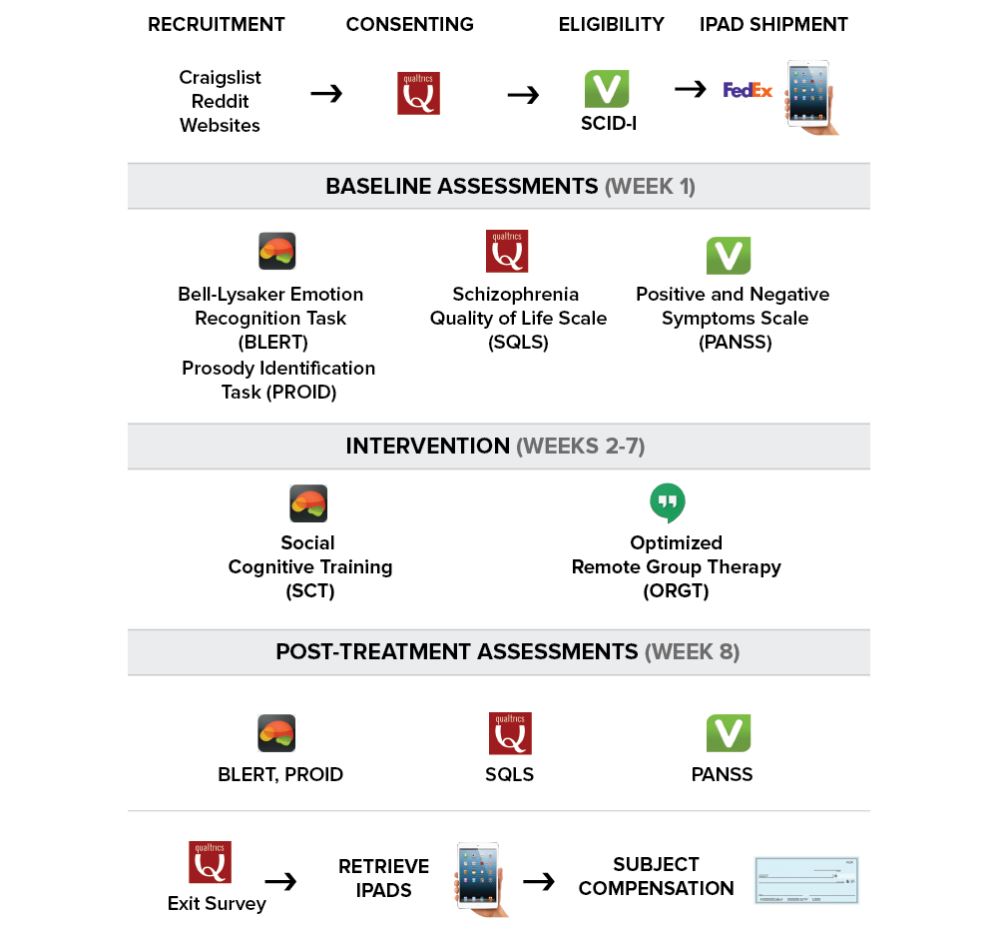
Creating Live Interactions to Mitigate Barriers (CLIMB) study procedures.

The study procedures are depicted in Figure 1. After enrollment, participants were given the opportunity to use their own iPad or to receive a study iPad via the mail that will be loaned to them for the duration of the trial. Study apps were preinstalled on the loaned iPads before shipment. Next, a one-on-one phone call was arranged to orient participants to the various apps. Enrolled participants were then placed on a waiting list and as soon as at least 3 individuals of similar age were enrolled and ready to participate in the trial, the cohort was assembled and underwent the assessment battery remotely using iPads. Once all cohort participants completed the assessment battery, they were engaged in the intervention for 6 weeks.

ORGT was delivered through Google Hangouts, a free platform that offers group-based videoconferencing, text chats and multimodal file sharing. At the beginning of the intervention, the moderator created a Hangouts group open to the clinician, moderator and cohort participants, and contacted the participants on the group text chat to explain her role. In the group text chat, the moderator introduced each participant to each other and to the clinician, informed them of the privacy practices in the apps and encouraged them to be respectful of their peers’ privacy. Next, the moderator invited participants to attend weekly, 60-minute group teletherapy sessions conducted through Google Hangouts video calls. In order to find a time that worked for everyone, the clinician, moderator and cohort participants indicated their availability using an online poll. During the 6 weeks of the intervention, the moderator was available online 8 hours a day, and kept the chat active in between sessions by contacting the group on a daily basis. Finally, all Hangouts interactions were archived and securely saved on Research Electronic Data Capture (REDCap), a HIPAA-compliant database.

The SCT program was delivered through the BrainHQ-Research app and was provided free of charge by Posit Science Inc. Participants were encouraged to complete 18 hours of SCT over the course of 6 weeks, preferably for 3 hours a week. In each training session, participants engaged with 4 different exercises, performing each exercise for about 7.5 minutes. To access the SCT program, participants logged into BrainHQ-Research using a unique study-provided login that contained no personally identifiable information. The moderator tracked user performance and treatment compliance remotely using a secure online portal. Weekly one-on-one phone calls were scheduled to discuss with participants their compliance with SCT requirements. Individualized motivational interviewing techniques were used when necessary to increase training frequency and structure training schedules [38]. For participants who completed less than 1 hour of SCT in the previous week, check ins were intensified up to 3 times a week. While in the intervention, participants continued to receive treatment by their outside providers (eg, psychoeducation, psychotherapy, adjustments in medications as clinically indicated).

Within 1 week after finishing the intervention, participants were asked to complete the assessment battery on iPads. Finally, they were asked to fill out an online exit survey in which they rated enjoyment, ease of use, perceived benefits and ease of fit into daily schedule.

If participants returned loaned iPads undamaged and fully functional, they were provided monetary compensation for participating in the study via a mailed check. Participants who completed all study procedures successfully earned US $285. Participants were paid US $5 for each completed hour of SCT and each ORGT video call attended, and US $15 per hour for assessments. Participants were not compensated for their participation in the group text chat.

Study participants were recruited online from various platforms and websites. After the phone screening, they signed informed consent documents digitally using Qualtrics. Next, they underwent a remote diagnostic evaluation (SCID), which was administered using the video-calling software Vsee. Once enrolled, participants received via mail an iPad, through which they completed an assessment battery. Next, they engaged in the CLIMB intervention for 6 weeks. CLIMB consists of a SCT program and ORGT. Within 1 week after finishing the intervention, participants completed the assessment battery on the iPad. Finally, they filled out an online exit survey. Upon the redelivery of the loaned iPad, they received compensation via check.

### Assessments

#### Social Cognition

We assessed social cognition by means of the Prosody Identification Task (PROID) [39], and the Bell-Lysaker Emotion Recognition Test (BLERT) [40], two well-validated computerized tasks. These tasks measure two of the constructs that were trained, but distinct and independent from the specific SCT exercises used in the intervention. PROID is a vocal identification task that assesses a subject’s ability to perceive and discriminate emotion in the speech of others. The test consists of 21 sentences of neutral content that are spoken aloud by male and female speakers to convey 1 of 7 different emotions (happiness, sadness, anger, fear, surprise, disgust and no emotion), as well as utterances with no emotional expressions. Participants first identify the emotional expression of each utterance and then rate the utterance's emotional intensity on a scale from 1 (very low intensity) to 9 (very high intensity). BLERT is an affect recognition task consisting of 21 short video clips in which an actor performs 1 of 3 dialogues while portraying 1 of 7 different emotions. Participants choose which of the 7 emotions listed on the screen best describes the affective quality enacted by the actor. For both tasks, trial-by-trial accuracy data are factorized for each emotion separately, and an overall accuracy score is also provided. iOS versions of BLERT and PROID were developed using original stimulus sets provided by the authors, embedded in the BrainHQ-Research app and administered without remote supervision. Alternate forms of PROID and BLERT were counterbalanced before and after the intervention.

#### Quality of Life

Quality of life was evaluated by means of the Schizophrenia Quality of Life Scale (SQLS) self-report questionnaire. SQLS is a 30-item questionnaire that requires a 7-day retrospective self-assessment of quality of life [41]. Results are scored using a 5-point Likert-type scale ranging from 1 (“Never”) to 5 (“Always”). Total score ranges from 30 (best status as measured on the SQLS) to 150 (the worst status as measured on the SQLS). The scale comprises the “Psychosocial,” “Motivation and Energy,” and “Symptoms and Side-Effects” subscales, with the purpose of indicating the extent of difficulty on each domain. The “Psychosocial” subscale (15 items) addresses various emotional problems, for example, feeling lonely, depressed or hopeless, as well as feelings of difficulty mixing in social situations and feeling worried about the future. The “Motivation and Energy” subscale (7 items) addresses various problems of motivation and activity, such as lacking the will to do things, while the “Symptoms and Side-Effects” subscale (8 items) addresses issues such as sleep disturbance, blurred vision, dizziness, muscle twitches and dry mouth, which can be caused by medications. The SQLS was digitized and completed without supervision through iPads using Qualtrics.

#### Clinical Symptoms

Clinical symptoms were assessed using the Positive and Negative Syndrome Scale (PANSS) [42]. From the PANSS scores, 6 symptom dimensions were derived: Positive, Negative, Disorganized, Excitement, Depression and Anxiety, and Other [43]. PANSS were conducted over Vsee by graduate-level psychology students trained on manual assessment procedures and observed by expert clinical supervisors. A large body of literature suggests that assessment via videoconferencing in patients with CPD is equivalent to in-person and is tolerated and well-accepted [44].

### Data Analysis Plan

To investigate the feasibility of CLIMB, descriptive statistics for recruitment, enrolment and retention rates, successful completion of remote assessments and iPad return rate were examined. Based on previous studies, we hypothesized that at least 75% of enrolled participants would complete the intervention [31], and that at least 85% of devices would be returned undamaged [45].

To investigate engagement in CLIMB, descriptive statistics for hours of SCT completed over the course of 6 weeks, attendance rate for group teletherapy and number of group chat messages and words were examined. Based on previous studies, we hypothesized that participants would complete at least 1 hour of SCT per week [31], participate in 80% of the group teletherapy sessions [22], and send at least 8 messages per week [30].

To investigate the acceptability of CLIMB, descriptive statistics from the CLIMB exit survey ratings for overall enjoyment, ease of use, ease of fit into daily schedule and perceived benefits were examined. We hypothesized ratings of at least 3 on the 5-point Likert scale items [31].

**Figure 2:**
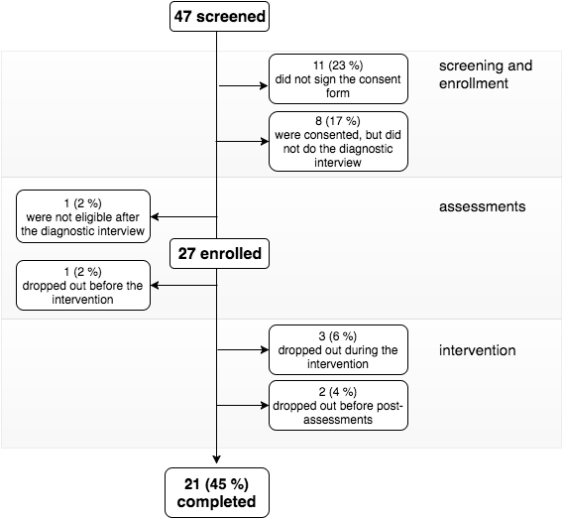
Consolidated Standards of Reporting Trials (CONSORT) flow diagram for Creating Live Interactions to Mitigate Barriers (CLIMB).

To explore the initial effects of CLIMB on study outcomes, we performed analyses on data obtained from all enrolled participants (N=27). Because we were interested in examining the ecological feasibility of CLIMB, we did not restrict the analyses only to participants who adhered to all intervention recommendations. Post-intervention data were not collected on 6 enrolled participants who dropped out at various stages of the intervention (see Figure 2). All outcome variables were normally distributed. Pre- to post-changes in outcome measures were examined using paired sample *t* tests. Within-group effect sizes (Cohen’s *d*) were computed using the mean change scores (post-treatment minus baseline) and the change score standard deviations. Because there were inter-individual differences in terms of engagement with each treatment component, and some engagement metrics were not normally distributed, we used non parametric correlations to test whether (1) engagement metrics were correlated; (2) demographic variables, symptoms or quality of life correlated with engagement metrics; and (3) changes in outcome scores correlated with engagement metrics. In the cases of significant pre- to post-changes and/or significant associations of these changes with engagement metrics, we used repeated measures linear mixed modeling with a diagonal covariance structure to determine whether changes in outcome measures were influenced by engagement with the intervention. Given the study attrition rate (22%), maximum likelihood (ML) estimation was used. Because we had baseline measurements for all participants, and the amount of missing data was relatively modest, it is likely that the missing at random assumption for ML was met, suggesting that it is unlikely the model results would have significantly changed had dropouts been able to be followed.

## Results

### Feasibility of Study Procedures

The Consolidated Standards of Reporting Trials (CONSORT) diagram of the study is shown in Figure 2. A total of 47 volunteer participants from 31 different states passed the phone screening over the course of 12 months (June 2015 to June 2016). Of these, 11 (23%, 11/47) never signed the consent form, 8 (17%, 8/47) were consented but did not participate in the SCID interview, and 1 (2%, 1/47) did not qualify after the SCID, leaving 27 participants enrolled in the study. Of these, 21 (78%, 21/27) completed all study procedures, 1 (3%, 1/27) dropped out during baseline assessments, 3 (11%, 3/27) dropped out during the intervention, and 2 (7%, 2/27) dropped out before post-treatment assessments. The reasons provided for dropping out included increased stress at work (n=1), symptom exacerbation (n=1), boredom (n=2), and unknown reasons (n=2). Over the course of 12 months, 7 cohorts were assembled.

**Figure 3:**
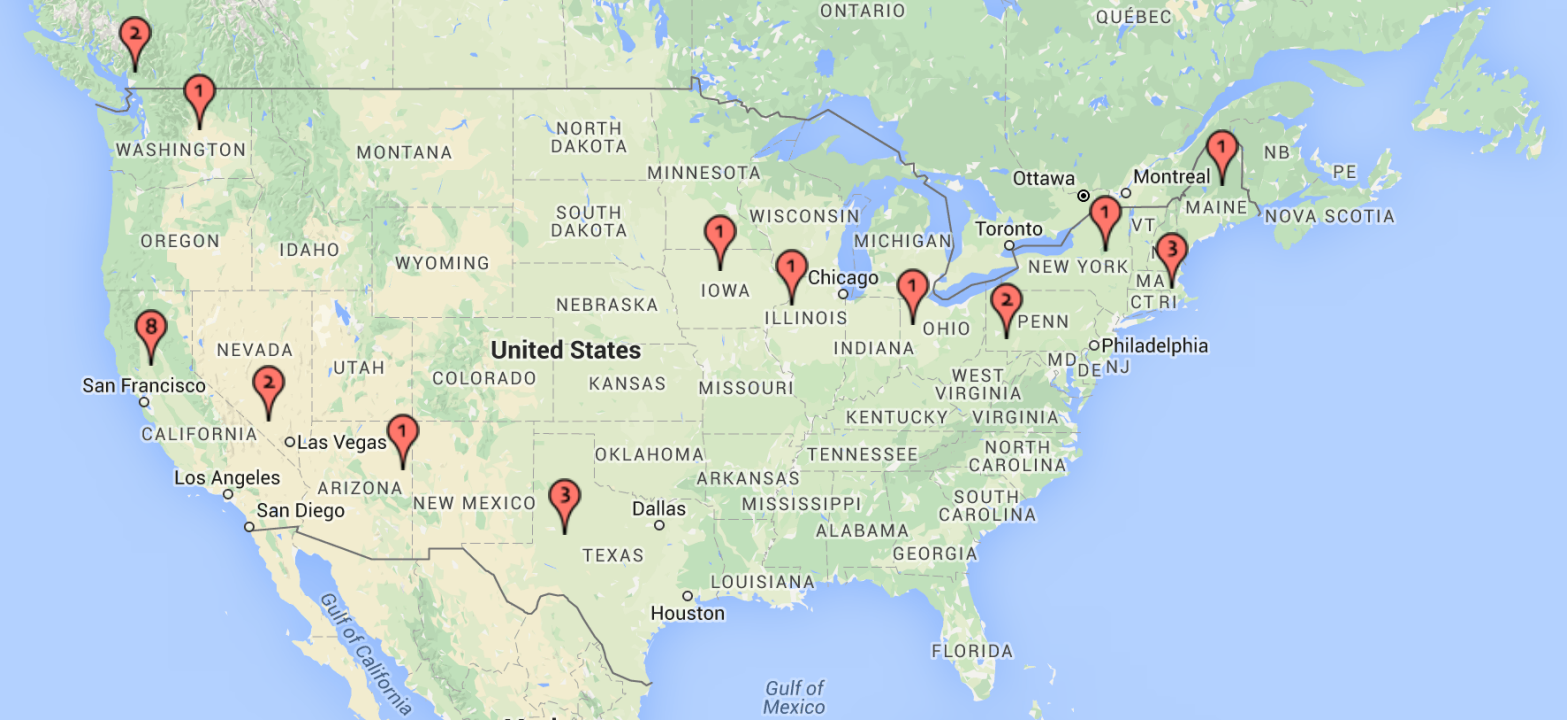
Geographic distribution of enrolled participants (N=27).

All phone-screened participants reported being comfortable using an iPad; 85% (40/47) had easy and regular access to wireless fidelity (WiFi), 72% (34/47) had a desktop or laptop computer in their home and 70% (33/47) had their own mobile phone with app capabilities (smartphone). The demographic information and access to mental health services for enrolled participants (N=27) is displayed in Table 1 and the geographic distribution of enrolled participants is shown in Figure 3.

**Table 1:** Demographic and baseline clinical and functional characteristics for the enrolled participants (N=27).

Characteristic		Mean (SD) or n (%)
**Sex, n (%)**		
	Female	10 (37%)
	Male	17 (63%)
Age (years), mean (SD)		28.1 (6.4)
Duration of illness (years), mean (SD)		7.0 (4.4)
Number of previous hospitalizations, mean (SD)		4.6 (4.0)
PANSS^a^ total score, mean (SD)		59.2 (15.6)
SQLS^b^ total score, mean (SD)		82.0 (15.9)
**Education, n (%)**		
	Drop-out in high school	1 (4%)
	High school degree	4 (15%)
	Currently pursuing a college degree	11 (41%)
	Drop-out during college	8 (30%)
	College degree	3 (11%)
**Medications, n (%)**		
	Taking antipsychotics	25 (93%)
	Unmedicated	2 (7%)
**Diagnosis, n (%)**		
	Schizophrenia	9 (33%)
	Schizoaffective	15 (56%)
	Bipolar disorder with psychosis	3 (11%)
**Access to mental health services, n (%)**		
	Seeing a psychiatrist	18 (67%)
	Seeing a case manager or nurse practitioner	7 (26%)
	Seeing a psychotherapist	7 (26%)

^a^PANSS: Positive and Negative Symptoms Scale.

^b^SQLS: Schizophrenia Quality of Life Scale.

Of the enrolled individuals, 4 (15%, 4/27) already had iPads. Consequently, iPads were shipped to 23 participants. Of those, 21 (91%, 21/23) were returned undamaged and fully functional, 1 (4%, 1/23) was initially withheld by an individual who dropped out of the study (and was retrieved with the help of the local police) and the final 1 (4%, 1/23) was never returned and was rendered unusable through remote deactivation. The administration of SCID interviews over Vsee was completed successfully with all enrolled participants. Of the enrolled individuals, 26 (96%, 26/27) completed the baseline assessment battery on iPads successfully, providing valid BLERT, PROID, SQLS and PANSS data.

### Engagement With Social Cognition Training and Optimized Remote Group Therapy

Participants were asked to complete 18 hours of SCT over the course of 6 weeks, but showed highly variable engagement, with a median of 9.5 hours of SCT completed (semi interquartile range of 6.3). In total, 6 participants (22%, 6/27) completed greater than 16 hours, requiring only brief weekly check ins; 8 (30%, 8/27) required regular weekly check ins and additional text reminders, and completed at least 6 hours; 8 (30%, 8/27) required intensive monitoring multiple times a week and trained 1 to 5 hours; and 4 (15%, 4/27) trained less than 1 hour and eventually dropped out, in spite of intensive monitoring. In addition, 46% (12/26) of participants completed less than 1 hour a week of training during the intervention. For a distribution of SCT hours, see Multimedia Appendix 2.

Participants attended on average 5.2 (SD 2.0) ORGT teletherapy sessions over the course of 6 weeks. Therefore, the average attendance rate for ORGT video calls was 84% (SD 28%). The participants’ self-assessment of social difficulties and preference about topics to be discussed during the group teletherapy sessions are shown in Multimedia Appendices 3 and 4. More than 40% of the participants endorsed lack of energy, social isolation, social and emotional withdrawal and general expectancy of failure. The topics that ranked as very interesting by at least 50% of the participants were, in order of preference, improving social engagement, improving speech activity, improving social cognitive skills, learning about identification of stressors and training problem solving skills.

Engagement with the ORGT group text chat was variable. Over the 6 weeks of the intervention, the total number of messages posted in the group chat by all cohort users (moderator and participants) averaged across all cohorts, was 1201 (SD 2013). The median number of messages posted by each participant per week across all participants, was 5.2, with a semi interquartile range of 12.8. The median number of words sent per participant per week was 37, with a semi interquartile range of 200. The median length of a message was 9.4 words, with a semi interquartile range of 5.1. We also calculated the ratio of moderator messages to participant messages. For instance, a ratio of 2:1 would mean that for every 2 messages sent from the moderator to participants in the group chat, each participant would post 1 message. When averaging across cohorts, we found a mean ratio of 0.97:1 (SD 0.30). The ratio reflects similar degrees of contribution to the group chat from the moderator and participants.

The attendance rate for group teletherapy positively correlated with hours of SCT completed (*r*=.484, *P*=.01), and with average number of words per message per participant (*r*=.44, *P*=.04). However, hours of SCT did not correlate with any ORGT messages and/or words metrics (all *P* values greater than .20).

At a qualitative level, we observed a wide range of contributions to ORGT: approximately 30% of participants were proactive during the group teletherapy sessions and sent text messages multiple times a week to the group chat, showing curiosity and appreciation, and engaging other participants in dynamic interactions; approximately 30% contributed only after encouragement from the clinician/moderator during the group teletherapy sessions and in the group text chat; and approximately 20% showed minimal contribution during the group teletherapy sessions, and left most engagement attempts in the group text chat unaddressed. Finally, original multimedia content was created by participants during the intervention and shared through Hangouts [46,47].

### Acceptability of Creating Live Interactions to Mitigate Barriers (CLIMB)

Upon study completion participants completed an online exit survey to rate their experience with CLIMB. The first component of the survey was a 23-item questionnaire that asked participants to indicate how much of the time they felt that each statement was true, using a 5-point Likert scale with 1 corresponding to “none of the time” and 5 to “all of the time.” Items were grouped into 4 categories, and the following averaged ratings were obtained: (1) Enjoyment/Satisfaction had a rating of 2.99 (SD 1.09), where 3 corresponds to half of the time; (2) Program Clarity/Ease of Use had a rating of 4.18 (SD 0.90); where 4 corresponds to most of the time; (3) Ease of Fit had a rating of 2.91 (SD 1.20); and (4) Perceived Benefits had a rating of 3.25 (SD 1.18). The complete list of items is included in Multimedia Appendix 5.

Finally, participants indicated what they liked best about the program and what kept them from adhering to CLIMB according to the recommended schedule. In summary, participants enjoyed participating in the ORGT group teletherapy sessions because of the positive, non-stigmatizing experience of social support from staff and peers during the sessions. They also appreciated being able to access the intervention from home, and speaking with other participants from a safe and protected environment. Internet technical difficulties, symptom exacerbation, low perceived value of the treatment, motivational deficits, employment burden and lack of time were self-reported as reasons for low engagement with CLIMB.

### Exploratory Outcomes

Significant improvements were found in pre- to post-measures of identification of vocal emotional prosody for happiness (*P*=.001) and happiness intensity (*P*=.04), as indexed by PROID (see Table 2). Participants also showed significant improvements in their ability to detect anger in a video vignette (*P*=.04), as indexed by BLERT. For these outcomes, effect sizes were large (*d*=.60 to *d*=.86). Trend-level improvements were observed on the SQLS total score, and “Psychosocial” (*P*=.09) and “Motivation and Energy” (*P*=.06) subscales. No significant changes were observed for PANSS ratings.

**Table 2:** Pre- to post-changes and effect sizes for outcome measures in study completers (N=21).

Outcome measure		Baseline, mean (SD)	Post, mean (SD)	Paired samples, *t* (sig)	Effect size, *d*
**PROID^a^ accuracy in detecting, %**					
	Happiness	0.35 (0.20)	0.54 (0.25)	–4.06 (0.00)	.86
	Happiness intensity	0.37 (0.18)	0.52 (0.27)	–2.26 (0.04)	.68
	Overall	0.58 (0.14)	0.59 (0.14)	–0.63 (0.54)	.11
**BLERT^b^ accuracy for detection (%)**					
	Anger	0.78 (0.30)	0.92 (0.15)	–2.26 (0.04)	.60
	Overall	0.72 (0.18)	0.80 (0.19)	–1.60 (0.13)	.42
**SQLS^c^**					
	Psychosocial	44.59 (9.58)	40.88 (10.71)	1.83 (0.09)	.36
	Symptoms and side effects	18.65 (5.01)	18.18 (4.49)	0.48 (0.64)	.10
	Motivation and energy	20.12 (3.55)	18.76 (3.70)	2.04 (0.06)	.37
	Total score	83.35 (16.22)	77.82 (17.14)	1.76 (0.10)	.33
**PANSS^d^**					
	Negative symptoms	2.33 (1.02)	2.63 (0.98)	–2.07 (0.09)	–.29
	Total score	60.81 (16.23)	61.05 (9.97)	–0.08 (0.94)	–.02

^a^PROID: Prosody Identification Task.

^b^BLERT: Bell-Lysaker Emotion Recognition Test.

^c^SQLS: Schizophrenia Quality of Life Scale.

^d^PANSS: Positive and Negative Symptoms Scale.

Less severe negative symptoms at baseline correlated with total number of words (*r*=–.497, *P*=.022) and messages (*r*=–.479, *P*=.028) posted per participant on the group text chat during the 6 weeks of the intervention. In addition, total number of words posted per participant on the group text chat correlated with shorter duration of illness (*r*=–.497, *P*=.022). Post-intervention outcomes were not inter-correlated. We found positive associations at trend level between hours of SCT completed and gains in total SQLS (*r*=.448, *P*=.071), SQLS “Psychosocial” (*r*=.455, *P*=.067) and SQLS “Motivation and Energy” (*r*=.460, *P*=.063). The results from the linear mixed models are shown in Multimedia Appendix 6. Hours of SCT did not have significant effects on the pre- to post-changes in the linear mixed models.

## Discussion

### Principal Findings

In this study, we tested the feasibility of CLIMB, a mobile psychosocial intervention designed to enhance social functioning in people with CPD. Using Internet-connected iPads, we delivered CLIMB for 6 weeks to people with CPD recruited remotely from 31 locations throughout the United States and Canada. We found that CLIMB is a highly feasible intervention with high enrolment, retention, iPad return and remote assessment completion rates. In particular, the attrition and device return rates in our study are similar to other studies testing mobile phone apps in patients with psychotic disorders [29,45]. Taken together, these findings indicate that delivering assessments and treatments remotely to people with CPD using mobile platforms is highly feasible.

In line with other mobile interventions for serious mental illness[48], engagement with the CLIMB treatment components was variable. The attendance rate for ORGT group teletherapy sessions was high (84%, SD 28%), and comparable to that of in-person group therapy approaches [22]. Despite significant efforts to monitor, support and keep participants engaged in SCT, 46% (12/26) of participants completed less than 1 hour a week of training during the intervention. One possible explanation of this differential engagement is that completion of SCT exercises requires sustained effort, focused attention and active planning and engagement by the user, whereas participation in ORGT may be less effortful, more directly rewarding and require less actively focused attention. Similar to previous studies [30], participants engaged in ORGT group text chatting multiple days a week. There were, nonetheless, inter-individual qualitative differences in terms of contribution such that some individuals engaged actively in text-based conversations with the group and shared original content (short videos, pictures, drawings, poems), while the majority of the users contributed marginally or minimally to the group text chat. This heterogeneity may partially be explained by the fact that contributions to the group text chat were not compensated. Baseline characteristics likely account for these inter-individual differences, where we found that participants with shorter duration of illness and fewer negative symptoms posted more words in the group text chat, though they did not complete more hours of SCT or showed higher attendance rate in the group teletherapy sessions. These findings suggest that the group text chat is a desired modality of social engagement particularly for individuals recently diagnosed with a psychotic disorder who have less severe negative symptoms. Interestingly, the higher attendance rate in ORGT group teletherapy sessions was associated with more hours of SCT and longer messages in the group text chat, whereas hours of SCT did not correlate with group text chat metrics, possibly suggesting that engagement in remote video calls had a positive effect on engagement in the other components of treatment.

The overall acceptability of CLIMB was medium, as reflected in the satisfaction ratings endorsed in the exit survey about ease of use and perceived benefits, as well as the retention rate. Participants valued the ease of use of the CLIMB apps, the ability to access the intervention from a safe and protected environment, and they felt comfortable and accepted when sharing subjective experiences with staff and other participants. Finally, 5 (19%, 5/27) participants provided unsolicited qualitative feedback about their participation (for representative excerpts, see Multimedia Appendix 7). Qualitative and quantitative data about acceptability collected from study participants in this pilot will inform future iterations on intervention development and optimization to meet the expectations and preferences of prospective service users.

We also examined the effect of 6 weeks of CLIMB on outcomes. We found significant pre- to post-intervention improvements on specific aspects of social cognition (prosody identification of happiness and recognition of anger) with large effect sizes, and trend-level improvements on quality of life self-reports, with medium effect sizes for the “Psychosocial” and “Motivation and Energy” subscales. Although there are currently no rigorous studies testing remote SCT in CPD, our within-group effect sizes for social cognition outcomes are comparable to recent reports of in-person SCT [16]. Improvements in quality of life self-reports showed trend-level relationships with hours of SCT completed. An appropriately powered, randomized controlled trial is required to determine whether CLIMB induces improvements in social cognitive outcomes, clinical symptoms, and quality of life.

### Limitations

Our study had several limitations. We recruited exclusively using the Internet, which most likely biases the sample, making it not representative of the larger population of individuals living with CPD. More than 70% of participants who passed the phone screening had a mobile phone, a desktop and/or laptop computer in their home and regular access to WiFi, whereas a recent meta-analysis found that mobile phone ownership among people with CPD was only 35% [49]. However, several lines of evidence now indicate that people with CPD already use their mobile devices to manage their illness and promote their recovery, and that mobile devices are an acceptable mental health method to deliver innovative interventions to people with CPD. For example, in surveys of mobile interventions acceptability, over half of the patients with CPD responded in favor of using mobile devices for tracking and/or monitoring their mental health, and for facilitating patient contact with health professionals [49]. As well, a large sample of individuals with CPD surveyed online reported that Web-based technology helped with identifying coping strategies and connecting for support with family and friends [50]. In addition, a convenient sample of young people recruited from two specialized early intervention programs for psychosis manifested interest in using the Internet, social media and mobile technologies for receiving mental health-related services [51]. Finally, a recent comprehensive review found high acceptability of delivered online and mobile interventions for serious mental illness, particularly when participants were provided remote online support, with the majority of studies reporting no effects of age, sex, educational level or clinical characteristics on acceptability [48]. While these findings provide evidence that acceptability is unlikely to represent a barrier to the implementation of CLIMB in mental health care settings, more problematic is the generalizability of the findings about intervention use, as less tech-oriented individuals with CPD may still find the approach acceptable, but not sufficiently engaging.

Two other factors contributed to the sampling bias. In order to be enrolled in the study, participants had to have awareness of their illness and be receiving mental health care. In our sample, 93% (25/27) of study participants were taking antipsychotic medications and 67% (18/27) were seeing a psychiatrist. These rates of mental health care use are much higher than the general CPD population in the United States, where at least 40% of people with CPD have no contact with mental health services [52]. As CLIMB has been designed to meet the needs of those who are unable or unwilling to come in to mental health clinics to access treatments for social functioning, future research will seek to recruit a mixed sample of service users directly drawn from diverse community-based settings and non-service users in order to examine engagement patterns on a more representative sample of patients with CPD. As inadequate engagement may detrimentally impact efficacy, dose-response studies will also be conducted to determine the necessary intervention duration and intensity to drive meaningful improvements in outcomes.

While it is true that the high recruitment and retention rates found in the study are likely the result of the sampling bias that may limit the generalizability of the findings and the ability to draw conclusions about the scalability potential of CLIMB, our sample nonetheless reflects a segment of younger individuals with CPD who are technology oriented, actively engaged in their recovery and treatment and for whom digital platforms seem to be a preferable intervention delivery modality [51].

Since the goal of this pilot study was to assess the feasibility of delivering CLIMB remotely and not its efficacy, we did not include a control group. We also did not place restrictions on medication regimens during study participation. Therefore, we cannot rule out non-specific effects of study participation and medication effects on the observed improvements on proximal measures of social cognition. In addition, the fact that participants were provided remuneration for each ORGT session attended and each hour of SCT completed, likely biased the data about engagement and adherence. Therefore, our results may not translate to real-world settings where this payment schedule may not be provided. Cost-effectiveness analyses and focus groups will be conducted to devise scalable solutions for sustained engagement that match users’ preferences and expectations. Finally, the current support protocol may be manageable for CLIMB moderators when serving small cohorts of patients, but may require significant adaptations to disseminate effectively.

### Conclusions

We demonstrated that it is feasible to target improvements in social cognition in people with CPD using an innovative and scalable treatment package that is delivered remotely, and that combines structured training of social cognitive abilities with a group-based, multimodal, ecological psychotherapeutic intervention. Future efficacy studies will evaluate the degree of improvement in various domains, including social functioning, social cognition, clinical symptoms and quality of life.

More importantly, results from this study indicate that it is feasible and acceptable to engage people with CPD in remote assessments and treatment using mobile devices. This has important implications in terms of access, engagement and dissemination of mental health services. First, providers will be able to interact with mobile interventions to monitor patient status remotely and provide inter-session extended support, at minimal costs and without requiring local infrastructures. Second, patients living in under-resourced areas who are unable or unwilling to come in to the clinic can benefit from specialized treatment options that may not be available locally. If successful, this approach has far-reaching implications for public health. As our knowledge of how to deliver effective treatments using remote digital technology grows, we will be able to reduce disparities in mental health outcomes, and promote equity in access to mental health care.

## Appendix

Multimedia Appendix 1 Descriptions of the social cognitive training (SCT) exercises.

Multimedia Appendix 2 Distribution of hours of social cognitive training completed over the course of 6 weeks (n=26). Each bar represents a Creating Live Interactions to Mitigate Barriers (CLIMB) participant.

Multimedia Appendix 3 Results from the online survey administered at baseline to assess current social difficulties.

Multimedia Appendix 4 Results from the online survey administered at baseline to review preferences for topics to be discussed during optimized remote group therapy (ORGT) sessions.

Multimedia Appendix 5 Quantitative data from the online exit survey. A 5-point Likert scale was used to determine how much of the time participants felt that each statement was true with 1 corresponding to none of the time, 2 to a little bit of the time, 3 to about half the time, 4 to most of the time, and 5 to all of the time (n=21).

Multimedia Appendix 6 Linear mixed models for outcome variables adjusting for hours of social cognition training (SCT) and using maximum likelihood (ML) estimation (N=27).

Multimedia Appendix 7 Excerpts of feedback provided by Creating Live Interactions to Mitigate Barriers (CLIMB) participants.

## Abbreviations

BLERT: Bell-Lysaker Emotion Recognition Test
CET: cognitive enhancement therapy
CLIMB: Creating Live Interactions to Mitigate Barriers
DSM-IV-TR: Statistical Manual of Mental Disorders-IV Text Revision
HIPAA: Health Insurance Portability and Accountability Act
IPT: integrated psychological therapy
ML: maximum likelihood
ORGT: optimized remote group therapy
PANSS: Positive and Negative Symptoms Scale
PROID: Prosody Identification Task
SCID: Structured Clinical Interview for the DSM-IV-TR
SCT: social cognition training
SMART: Specific, Meaningful, Agreed Upon, Realistic, Timely
SQLS: Schizophrenia Quality of Life Scale
WiFi: wireless fidelity
