# Supplementary material for: Creating Live Interactions to Mitigate Barriers (CLIMB): A Mobile Intervention to Improve Social Functioning in People With Chronic Psychotic Disorders
Source: JMIR Ment Health. 2016 Dec 13;3(4):e52. doi: 10.2196/mental.6671 (PMC5192235; doi:10.2196/mental.6671)
Supplement: Multimedia Appendix 1 [file mental_v3i4e52_app1.pdf]

Descriptions of the social cognitive training (SCT) exercises:

1. Eye Gaze Matching: this exercise is designed to improve processing speed for accurate identification of the direction of eye gaze. Each trial starts with a central 'start' button. Once the user clicks on the start button, a target face is presented for a brief period of time, looking at one of 9 potential directions (randomly selected with equal probability) followed by a visual mask for 500ms, and then an array of between 2 and 9 faces. Participants are required to select the face where the eyes are gazing in the same direction as the target face (regardless of face identity; Figure 1, middle panel) by clicking on it with the computer mouse. Auditory feedback is provided for both correct and incorrect responses, and the next trial begins 3 s after the participant's response. In this exercise, the duration of presentation of the target face is adaptively varied based on participant's responses using a Zest algorithm (King-Smith et al., 1994), which is a Bayesian adaptive psychometric method that uses maximum likelihood procedure for threshold estimation. Each exercise block contains 50 trials.

2. Face Matching: the purpose of this exercise is to improve processing of facial features. The structure of this exercise is similar to that of S Gaze Match (see above). On each trial, a face is presented (randomly selected from a database of 100 faces) for a brief period of time, from either the front, the side, or three-quarters angle. The target face is followed by a visual mask, and then an array of between 2 and 12 faces (depending on the level). Participants are required to select the target face from the array (note that on more difficult levels the face in the array can be rotated compared to the target). Adaptivity and threshold calculation is done using 1up-2down procedure (Levitt, 1971), with a step size of 50ms, converging to 71% correct.

3. Face Emotion Match (Speeded Facial Emotion Matching): the purpose of this exercise is to improve the brain's ability to make implicit speeded decisions about facial emotion features. The exercise is similar in structure to the Eye Gaze Matchubg exercise, but here the target face features an emotion and the response array features 2-8 faces (depending on difficulty) each showing a different emotion. Feedback, adaptivity, and threshold calculation are done similarly to the S Gaze Match exercise.

4. Facial Emotion CPT (Facial Emotion Continuous Performance Task): this exercise aims to improve the brain's ability to distinguish between emotionally expressive faces and neutral faces. The user is instructed to press the spacebar on his or her keyboard when shown an image of a smiling (80% occurrence) or frowning (10% occurrence) face but to withhold such action in response to a neutral face (10% occurrence). The images in this task are of male and female individuals from children to adults. Inter-stimulus-interval is randomly selected to be either 600, 1800 or, 3000ms, with equal probability. The user is instructed to respond as quickly as possible to an emotional image A block of the task consists of 240 trials.

5. Face Stories Span: The purpose of this exercise is to strengthen memory span for social facts. On every trial, pictures of individuals are presented along with three facts about each individual (e.g. 'George like to eat pasta'). The user is then prompted with the faces in random order, and should select the correct fact, out of 3, about that person. In a successful trial the user should correctly select all facts presented (one per person). The length of the sequence is adaptively set using a 2up-1down adaptive rule. A block is comprised of 15 trials.

6. Vocal Emotion Id (Vocal Emotion Identification): The purpose of this exercise is to strengthen the ability to understand prosody cues in speech. Every trial starts with a presentation of a 'start' button.

After clicking it, there is a 1000ms delay which is followed by a sentence with neutral content (e.g. ‘Today is Tuesday’) but spoken with emotional prosody, such as a happy voice. The sentence is followed by 2-5 verbal emotion labels, the target emotion and distractor emotion labels. The participant must select the emotion label which matches the emotional prosody of the sentence. The target emotion is randomly selected with equal probability from the following five basic emotions: neutral, happy, sad, angry and afraid. The length of the target sentence is adaptively changed between short (1-2 words), medium (3-4 words) and long (5-7 words) based on a 2down-1up adaptive rule (Levitt, 1971), where the sentence gets longer if the participant makes a mistake and gets shorter if the participant is correct two consecutive times. Threshold is calculated as the arithmetic mean of last 5 reversals. The total number of trials in an assessment block is 35.

8

7. Vocal Affect Theory of Mind (ToM) : This exercise is designed to improve the ability to extract ToM cues from vocal variations. On every trial, the user hears a short script describing a social situation. The user is then prompted with a question about the script, for example ‘what would Sally sound like in this situation?’ Then, three response options appear. The three options correspond to the same sentence spoken by the character, every time with a different prosody (e.g. to reflect angry, excited, or happy mood). The user should select the sentence with the prosody that matches that of the character based on the script. The length of the response sentences is varied based on the user responses (longer sentences are played if the user makes mistakes).
