# Supplementary figures and images for: Creating Live Interactions to Mitigate Barriers (CLIMB): A Mobile Intervention to Improve Social Functioning in People With Chronic Psychotic Disorders
Source: JMIR Ment Health. 2016 Dec 13;3(4):e52. doi: 10.2196/mental.6671 (PMC5192235; doi:10.2196/mental.6671)

Hours of Social Cognition Training  
(SCTI) completed after 6 weeks

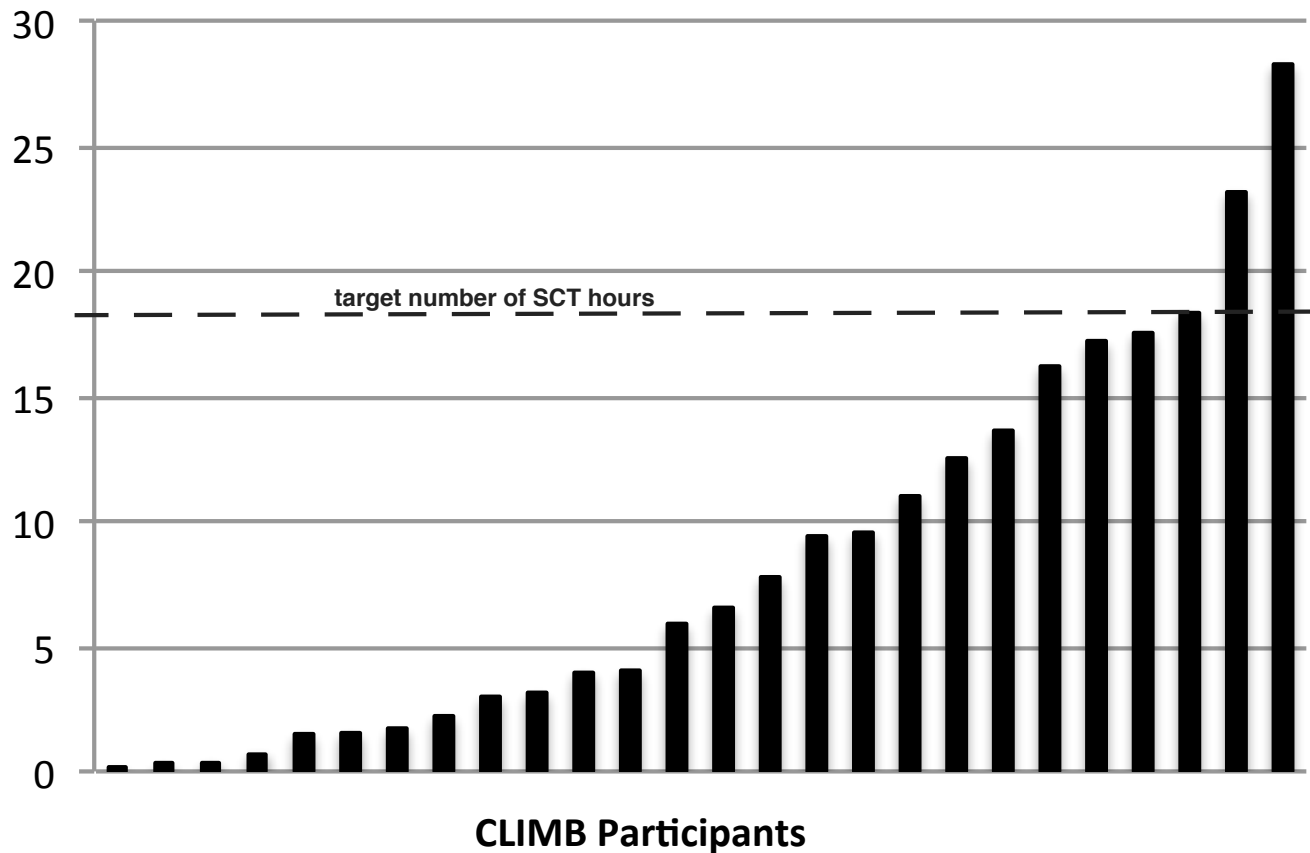

Supplement: Multimedia Appendix 2 [file mental_v3i4e52_app2.pdf]
