# Supplementary material for: Creating Live Interactions to Mitigate Barriers (CLIMB): A Mobile Intervention to Improve Social Functioning in People With Chronic Psychotic Disorders
Source: JMIR Ment Health. 2016 Dec 13;3(4):e52. doi: 10.2196/mental.6671 (PMC5192235; doi:10.2196/mental.6671)
Supplement: Multimedia Appendix 3 [file mental_v3i4e52_app3.pdf]

**How Often do you experience:**	Never (%)	Not very often (%)	Sometimes (%)	Most of the time(%)	All the time (%)
discomfort in social situations?	0.0	9.5	52.4	14.3	23.8
lack of energy?	0.0	13.6	36.4	27.3	22.7
difficulties with emotion expression?	0.0	22.7	50.0	4.6	22.7
difficulties with emotion detection?	9.5	23.8	28.6	23.8	14.3
social isolation?	0.0	22.7	36.4	27.3	13.6
social and emotional withdrawal?	4.6	18.2	36.4	31.8	9.1
perception of limited resource?	9.1	22.7	40.9	18.2	9.1
difficulties with perception of social situations?	9.5	19.1	42.9	23.8	4.8
lack of motivation?	0.0	13.6	54.6	27.3	4.6
lack of initiative/planning?	0.0	31.8	45.5	18.2	4.6
generalized expectancy of failure?	13.6	18.2	27.3	40.9	0.0
low self-esteem?	4.6	9.1	54.6	31.8	0.0
demoralization?	27.3	22.7	22.7	27.3	0.0
low self-worth?	18.2	22.7	40.9	18.2	0.0
hopelessness?	22.7	27.3	31.8	18.2	0.0
