# Supplementary material for: Creating Live Interactions to Mitigate Barriers (CLIMB): A Mobile Intervention to Improve Social Functioning in People With Chronic Psychotic Disorders
Source: JMIR Ment Health. 2016 Dec 13;3(4):e52. doi: 10.2196/mental.6671 (PMC5192235; doi:10.2196/mental.6671)
Supplement: Multimedia Appendix 4 [file mental_v3i4e52_app4.pdf]

How interested would you be in:	Not interested (%)	Indifferent (%)	Somewhat interested (%)	Very interested (%)
improving social engagement?	9.1	0.0	31.8	59.1
improving speech activity?	4.6	4.6	36.4	54.6
improving social cognitive skills, like emotion detection and expression?	9.1	13.6	27.3	50.0
learning about identification of stressors?	13.6	9.1	27.3	50.0
training your problem-solving skills?	4.6	27.3	18.2	50.0
reducing a generalized expectancy of failure ?	4.8	14.3	33.3	47.6
managing mood swings?	13.6	13.6	27.3	45.5
managing social anxiety?	9.1	18.2	27.3	45.5
learning about relaxation techniques?	13.6	18.2	22.7	45.5
managing hallucinations and paranoia?	18.2	13.6	27.3	40.9
improving emotional participation?	0.0	18.2	50.0	31.8
developing activity scheduling skills?	9.1	22.7	36.4	31.8
developing initiative/planning skills?	14.3	9.5	47.6	28.6
managing anger ?	4.6	27.3	40.9	27.3
building self-esteem?	4.6	18.2	54.6	22.7
