# Supplementary material for: Creating Live Interactions to Mitigate Barriers (CLIMB): A Mobile Intervention to Improve Social Functioning in People With Chronic Psychotic Disorders
Source: JMIR Ment Health. 2016 Dec 13;3(4):e52. doi: 10.2196/mental.6671 (PMC5192235; doi:10.2196/mental.6671)
Supplement: Multimedia Appendix 5 [file mental_v3i4e52_app5.pdf]

	MEAN	SD
**Enjoyment/ Satisfaction**	**2.99**	**1.09**
CLIMB is interesting?	3.00	1.06
CLIMB is fun?	2.82	0.95
CLIMB is satisfying?	3.24	1.20
CLIMB is enjoyable?	2.71	0.99
you would recommend CLIMB to others?	3.18	1.24
**Program Clarity / Ease of use**	**4.18**	**0.90**
It's easy to use the iPad?	4.65	0.49
CLIMB makes it quick and easy for you to train every day?	3.53	1.18
CLIMB apps are easy to navigate?	4.18	0.95
CLIMB instructions are easy to understand?	4.35	0.93
CLIMB conveniently helps you remember where you last left off in your training session?	4.18	0.95
**Ease of fit into daily schedule**	**2.91**	**1.20**
CLIMB fits easily into your daily schedule?	3.00	1.22
it's easy to do CLIMB 4 hours per week?	2.24	1.30
the one hour remote session passes by quickly?	3.94	1.12
CLIMB is a high priority, compared to your other activities	2.65	1.17
you would use CLIMB even if you were not part of the study?	2.71	1.21
**Perceived benefits**	**3.25**	**1.18**
you are making progress?	3.59	1.06
CLIMB session feels helpful to you?	3.29	1.16
CLIMB is effective?	3.24	1.15
CLIMB will improve your thinking and memory?	3.12	1.22
CLIMB will help you do well at school or at work?	2.94	1.20
CLIMB is having a positive effect on your daily life?	3.29	1.31
