# Supplementary material for: Creating Live Interactions to Mitigate Barriers (CLIMB): A Mobile Intervention to Improve Social Functioning in People With Chronic Psychotic Disorders
Source: JMIR Ment Health. 2016 Dec 13;3(4):e52. doi: 10.2196/mental.6671 (PMC5192235; doi:10.2196/mental.6671)
Supplement: Multimedia Appendix 6 [file mental_v3i4e52_app6.pdf]

		Mixed Models			
		Effects of Time		Effects of SCT hours	
		F	P	F	P
PROID Accuracy in Detecting (%)	Happiness	10.08	**0.00**	0.91	0.35
	Happiness Intensity	5.67	**0.02**	0.88	0.36
	Overall	0.27	0.61	7.21	**0.01**
BLERT Accuracy for Detection (%)	Anger	3.42	**0.07**	0.01	0.91
	Overall	2.21	0.15	0.99	0.33
Quality of Life	Psychosocial	0.57	0.45	0.32	0.58
	Symptoms & Side Effects	0.02	0.89	0.21	0.65
	Motivation & Energy	1.18	0.28	0.11	0.74
	Total Score	0.55	0.47	0.05	0.83
PANSS	Negative Symptoms	1.78	0.19	0.10	0.76
	Total Score	0.03	0.86	1.13	0.29
