# Supplementary material for: Creating Live Interactions to Mitigate Barriers (CLIMB): A Mobile Intervention to Improve Social Functioning in People With Chronic Psychotic Disorders
Source: JMIR Ment Health. 2016 Dec 13;3(4):e52. doi: 10.2196/mental.6671 (PMC5192235; doi:10.2196/mental.6671)
Supplement: Multimedia Appendix 7 [file mental_v3i4e52_app7.pdf]

Excerpts of feedback provided by CLIMB participants.

*"I was not sure what to expect, I was definitely imagining something very clinical and rigid, but I was ok with that. Bit by bit as I was interviewed I noticed the process was more human and organic and real"*

*"I felt very positive and affirmed. I liked hearing them affirm that they believe in me and think I'm doing great for my situation. It really really helps to be affirmed that way. It would be very very detrimental if I was being told to change or that I am sick or all these infertile ideas like most doctors tend to communicate. I feel very encouraged and inspired. I feel safe and fortunate and connected"*

*"After a few weeks I began to notice similarities between the expressions I saw in the faces of other people in my life and qualities to their voices that reminded me of the app. I was better able to identify the emotions of my surrounding humans and accomplish more successful social interactions."*

*"I shall mention the benefits of the combined work with brain training and the group therapy. Had we merely been completing brain training and lacking in the group therapy, I do not believe the brain training would have been so successful. Similarly, I do not believe the group therapy would have been as successful were we not attempting the brain training. I find this the case because the brain training provided us with a mutual experience but also exercises that improved our social cognition. The group therapy, in turn, allowed us to experience social interaction, address issues that we were sharing, and to apply concretely the gifts of the training through social interaction."*

*"My experience with the training was pleasant and valuable. It taught me to notice things that normally I would not notice. The process of learning was easy and fun. I stayed engaged in the whole learning process, my efforts seems like they would pay off".*

*"At times of late I struggle socially, and sometimes felt anxious about doing the group therapy. Though these feelings were often forgotten as the group setting felt reasonably comfortable and full of acceptance; from participants and the 2 staff members involved with the study."*

*"I liked being treated like a unique person instead of a sick patient. I liked sharing art. It was very happy to look forward too. I loved the feedback. I loved the things we could express through our art that we could not express the same way with just conversation."*
